# Complex DNA Damage Induced by High Linear Energy Transfer Alpha-Particles and Protons Triggers a Specific Cellular DNA Damage Response

**DOI:** 10.1016/j.ijrobp.2017.11.012

**Published:** 2018-03-01

**Authors:** Rachel J. Carter, Catherine M. Nickson, James M. Thompson, Andrzej Kacperek, Mark A. Hill, Jason L. Parsons

**Affiliations:** ∗ Cancer Research Centre, Department of Molecular and Clinical Cancer Medicine, University of Liverpool, United Kingdom; † Gray Laboratories, Cancer Research UK/Medical Research Council Oxford Institute for Radiation Oncology, University of Oxford, Oxford, United Kingdom; ‡ The National Eye Proton Therapy Centre, The Clatterbridge Cancer Centre NHS Foundation Trust, Bebington, United Kingdom

## Abstract

**Purpose:**

To investigate the precise mechanism of recognition and processing of ionizing radiation (IR)-induced complex DNA damage (CDD), where two or more DNA lesions are in close proximity, in cellular DNA which is packaged with histones to form chromatin.

**Methods and Materials:**

HeLa and oropharyngeal squamous cell carcinoma (UMSCC74A and UMSCC6) cells were irradiated with high linear energy transfer (LET) α-particles or protons, versus low-LET protons and X rays. At various time points after irradiation, site-specific histone post-translational modifications were analyzed by quantitative Western blotting; DNA damage and repair were measured by different versions of the comet assay; and cell survival was determined using clonogenic assays.

**Results:**

Site-specific histone post-translational modifications after low- and high-LET radiation, particularly proton irradiation, were screened, aiming to identify those responsive to CDD. We demonstrate that histone H2B ubiquitylated on lysine 120 (H2B_ub_) is specifically induced several hours after irradiation in response to high-LET α-particles and protons but not by low-LET protons or X rays/γ-radiation. This is associated with increased levels of CDD, which contributes to decreased cell survival. We further discovered that modulation of H2B_ub_ is under the control of two E3 ubiquitin ligases, MSL2 and RNF20/RNF40 complex, whose depletion leads to defective processing and further persistence of CDD, and to additional decreased cell survival after irradiation.

**Conclusion:**

This study demonstrates that the signaling and repair of CDD, particularly induced by high-LET IR is co-ordinated through the specific induction of H2B_ub_ catalyzed by MSL2 and RNF20/40, a mechanism that contributes significantly to cell survival after irradiation.

**Summary**

Complex DNA damage (CDD), a signature of ionizing radiation, contributes significantly to cell killing, although the precise mechanism of recognition and processing in cellular DNA is currently unknown. By comparing cellular effects of low versus high linear energy transfer radiation, therefore varying CDD frequency, we demonstrate that histone H2B ubiquitylated on lysine 120 catalyzed by MSL2 and RNF20/RNF40 complex is essential for CDD repair after high-linear energy transfer α-particles and protons and in controlling cellular radiosensitivity.

## Introduction

Ionizing radiation (IR) induces a plethora of different types of DNA damage, of which DNA double-strand breaks (DSBs) are a relatively small proportion (<5%) and where DNA single-strand breaks (SSBs) and DNA base damage predominate. However, IR deposits energy in highly structured tracks, which can lead to the formation of complex DNA damage (CDD; also termed clustered DNA damage), defined as two or more DNA lesions induced in close proximity (ie, within 1 to 2 helical turns of the DNA). Because of their complex nature, CDD and DSBs are thought to be the major contributors to IR-induced cell killing and therefore important in the therapeutic effect of radiotherapy in cancer treatment. This is because non-DSB CDD has been shown to be difficult to repair both in vitro using synthetic oligonucleotide substrates and also in bacterial, yeast, and mammalian cells using plasmid reporter systems 1, 2. Consequently, whereas SSBs have a short lifetime (<30 minutes) and DSBs have an intermediate lifetime (approximately 1-2 hours), CDD can persist in cells and tissues several hours after IR 3, 4. Mathematical modeling has estimated that CDD increases from approximately 30% for low linear energy transfer (LET) IR up to 90% for high-LET α-particles (α-IR) 5, 6, 7, 8. Proton beam irradiation, which is increasingly being used therapeutically for cancers, including head and neck cancers (9), has also been predicted to generate CDD, with the proportion dependent on energy and LET. In particular, high-energy (low-LET) protons are thought to generate a DNA damage spectrum similar to that of X rays and γ-irradiation (γ-IR), whereas low-energy protons (with increased LET), specifically at the Bragg peak distal end, generate CDD with increasing frequency. Indirectly, this has been shown by demonstrating that increasing proton LET leads to decreased cell survival and an increase in persistent DNA DSBs 10, 11, 12. This suggests that protons at specific energies are more effective at killing cancer cells through CDD formation and should be considered in developing optimal therapeutic strategies. Despite this, the cellular response to CDD particularly induced by high-LET α-IR and protons is currently unclear.

Nucleosomes form the basic structure of chromatin, where DNA is wrapped around a histone octamer consisting of two copies each of the core histone proteins (H2A, H2B, H3 and H4). The N-terminal tails of histones can be subject to posttranslational modifications (PTMs), including acetylation, methylation, phosphorylation, and ubiquitylation, which can dynamically alter chromatin structure and are important for several DNA-dependent processes, including DNA repair (13). The most well-characterized modification induced by DNA damage is the phosphorylation of the histone variant H2AX on serine 139 (also known as γH2AX [14]) at sites of DNA DSBs catalyzed by ataxia telangiectasia mutated (ATM), but also ATM and Rad3 related (ATR), and DNA-dependent protein kinase (DNA-Pk). Formation of γH2AX causes recruitment of DNA damage response proteins, such as MDC1 and 53BP1, and also stimulates localization of chromatin modifiers to the DSB site, largely through a ubiquitylation-dependent process (15). Indeed, E3 ubiquitin ligases and deubiquitylation enzymes control H2A and H2AX ubiquitylation and, in association with chromatin remodeling complexes, assist in DSB repair. However, other histone modifications, including induction of H2B ubiquitylation at lysine 120 by RNF20/RNF40 (16), which may also impact on H3 methylation on lysine 4 (17), and a reduction in H3 acetylation at lysines 9 and 56 (18) are also thought to be modulated at DSB sites. Collectively this highlights the importance of histone PTMs in the cellular DNA damage response by promoting chromatin relaxation and coordinating recruitment of DNA damage repair proteins. However, the site-specific histone PTMs, as well as the enzymes and mechanisms involved in the signaling and processing of CDD, are currently unknown and are required for improving our understanding of radiobiology.

## Methods and Materials

### Antibodies, proteins, and small interfering RNA

Primary/secondary antibodies, proteins, and small interfering RNA (siRNA) sequences can be found in the Supplementary Information (available online at www.redjournal.org).

### Cell culture

HeLa, UMSCC6, and UMSCC74A cells were cultured under standard conditions as previously described (19). siRNA knockdowns were performed for 48 hours using Lipofectamine RNAiMAX (Life Technologies, Paisley, United Kingdom).

### Irradiation sources

Cells were exposed to low-LET γ-IR using a ^137^Cs sealed-source irradiator (GSR D1, Gamma-Service Medical, Leipzig, Germany; dose rate of 2.0 Gy/min). Cells were exposed to low-LET X rays (100 kV) using CellRad x-ray irradiation (Faxitron Bioptics, Tucson, AZ; dose rate of 3 Gy/min). Proton irradiations were performed using a horizontal, passive-scattered beam line of 60 MeV maximal energy from the Douglas Cyclotron at Clatterbridge (20). Cells in 35-mm dishes were positioned at the isocenter 70 mm from a brass collimator (43 mm diameter). For high-energy protons, cells were irradiated directly by an approximately 1 keV/μm pristine beam of 58 MeV effective energy (dose rate of approximately 5 Gy/min). Low-energy proton irradiations were performed using a modulator to generate a 27 mm spread-out Bragg peak. A 24.4 mm Perspex absorber was used to position the cells at the distal edge of the spread-out Bragg peak, corresponding to a mean proton energy of 11 MeV at a dose-averaged LET of 12 keV/μm (dose rate of approximately 5 Gy/min). Cells were irradiated with 3.26 MeV α-IR (LET of 121 keV/μm; dose rate of approximately 1.2 Gy/min) using a ^238^Pu irradiator. Cell cycle analysis was performed by fluorescence-activated cell sorting (FACS; Supplementary Information, available online at www.redjournal.org).

### Histone extractions and Western blotting

Cells were irradiated with 10 Gy X rays, α-IR, or protons or treated with 150 μM H_2_O_2_ for 15 minutes and harvested by centrifugation (1500 rpm, 5 minutes, 4°C). To inhibit DNA transcription, cells were preincubated for 1 hour with 1 μg/mL actinomycin D before irradiation. Histones were purified by acid extraction, as previously described (21), and analyzed by quantitative Western blotting using the Odyssey image analysis system (Li-cor Biosciences, Cambridge, United Kingdom) as described in the Supplementary Information (available online at www.redjournal.org) from at least 3 independent experiments.

### Single cell gel electrophoresis (comet) assays

The alkaline comet assay for measurement of SSBs and alkali labile sites and the neutral comet assay for DSBs have been previously described 19, 22, 23. For detection of CDD, an enzyme-modified neutral comet assay was used similar to that previously described (24) but with modifications as described in the Supplementary Information (available online at www.redjournal.org). Percent tail DNA values were calculated from at least 3 independent experiments and normalized against those seen immediately after irradiation, which were set to 100% to allow for comparative kinetics of DNA repair. However, for detection and revealing CDD sites, absolute values of percent tail DNA are shown.

### Clonogenic assays

After irradiation in 35 mm dishes, cells were trypsinized and counted and a defined number seeded in triplicate into 6 well plates (250/500 for HeLa and 2000/4000 for UMSCC74A/UMSCC6 for unirradiated controls). Plating efficiencies for HeLa and UMSCC74A/UMSCC6 were approximately 40% and 5%, respectively. Note that increasing cell numbers were used for increasing doses of IR, and double or quadruple the numbers of cells were plated following MSL2 and RNF20 siRNA, respectively, to account for plating efficiencies. Colonies were allowed to grow (approximately 7-10 days) before fixing and staining with 6% glutaraldehyde, 0.5% crystal violet for 30 minutes. Colonies were counted using the GelCount colony analyzer (Oxford Optronics, Oxford, United Kingdom). Surviving fraction (SF) was expressed as colonies per treatment versus colonies in the untreated control from at least 3 independent experiments. Data were fitted to the equation ln(SF) = −α*D*, where *D* equals dose using OriginPro 9.1, and the relative biological effectiveness (RBE) with associated errors was calculated relative to high-energy protons by determining the proton doses required to give a surviving fraction of 0.5.

## Results

### H2B_ub_ is induced in response to IR-induced CDD

**Fig. 1:**
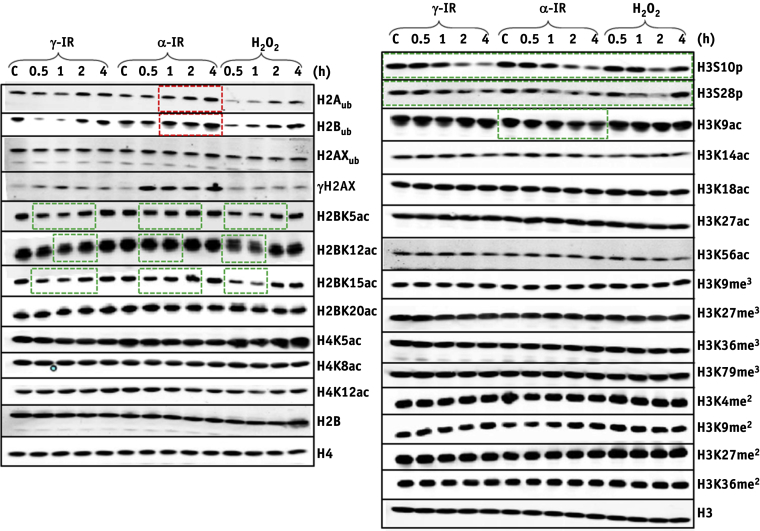
Analysis of histone post-translational modifications (PTMs) in response to γ-irradiation, α-particles, and hydrogen peroxide. HeLa cells were untreated (designated C), irradiated with 10 Gy γ-irradiation/α-particles or treated with 150 μM H_2_O_2_, and harvested at the time points indicated after treatment and purified histones analyzed by immunoblotting. Red boxes indicate increased levels of histone PTMs, whereas green boxes indicate reduced levels. Shown are representative images acquired from at least two independent experiments, and levels of the respective unmodified histone are shown as a loading control. (A color version of this figure is available at www.redjournal.org.)

**Fig. 2:**
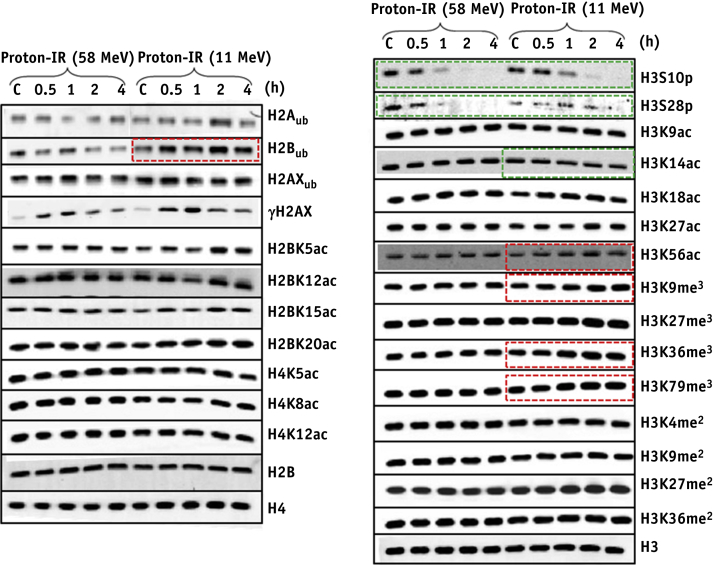
Analysis of histone post-translational modifications (PTMs) in response to proton irradiation. HeLa cells were untreated (designated C) or irradiated with 10 Gy protons at low or high linear energy transfer and harvested at the time points indicated after treatment and purified histones analyzed by immunoblotting. Red boxes indicate increased levels of histone PTMs, whereas green boxes indicate reduced levels. Shown are representative images acquired from at least two independent experiments, and levels of the respective unmodified histone are shown as a loading control. (A color version of this figure is available at www.redjournal.org.)

HeLa cells were irradiated with low-LET γ-IR or high-LET α-IR known to generate CDD in different proportions, and histone PTMs were analyzed at various time points after treatment. Cells were also treated with hydrogen peroxide. The focus was on identifying specific histone PTMs responsive to CDD induced by α-IR, particularly at later time points after IR where CDD persists. Reductions in H3S10 and H3S28 phosphorylation (mitotic markers) and in acetylation of H2B were observed irrespective of the treatment. H2B ubiquitylation at lysine 120 (H2B_ub_) increased at 1 to 4 hours after treatment following α-IR, whereas levels initially decreased and then returned to control levels following γ-IR and hydrogen peroxide (Fig. 1). Using low-energy (11 MeV; relatively high-LET) and high-energy (58 MeV; low-LET) protons, a reduction in H3S10 and H3S28 phosphorylation was also observed (Fig. 2). Additionally, no difference in cell cycle position following low- or high-LET protons was observed (Fig. E1A and E1B, available online at www.redjournal.org). High-LET protons appear to cause elevations in the levels of H3K56 acetylation and H3 trimethylation. However, similar to α-IR, induction of H2B_ub_ was observed at later time points after IR where CDD persists.

**Fig. 3:**
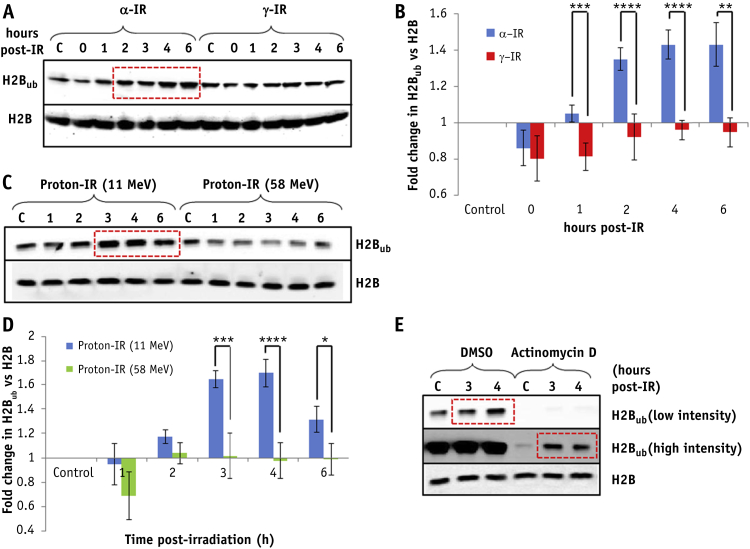
H2B_ub_ is induced in HeLa cells in response to complex DNA damage induced by high linear energy transfer (LET) α-particles and protons. (A-D) HeLa cells were untreated (designated C), irradiated with 10 Gy γ-irradiation, α-particles, or protons at low or high LET, and harvested at the time points indicated after ionizing radiation and purified histones analyzed by immunoblotting. In (E), cells were pretreated with dimethyl sulfoxide (DMSO) or actinomycin D before irradiation with 10 Gy high-LET protons. Red boxes indicate increased levels of H2B_ub_. (B, D) Shown is the mean fold change ± standard deviation in H2B_ub_ normalized against H2B. *P < .05, **P < .01, ***P < .005, ****P < .002, as analyzed by a two-sample *t* test.

Induction of H2B_ub_ was confirmed quantitatively following α-IR and high-LET protons from 2 hours after IR, versus γ-IR and low-LET protons, and this induction was maintained 3 to 6 hours after IR where CDD persists (Fig. 3A-D). To exclude that this was associated with transcription, cells were preincubated with actinomycin D. Using dimethyl sulfoxide as a control, there was still a significant accumulation of H2B_ub_ at 3 to 4 hours after treatment with high-LET protons versus the unirradiated control (Fig. 3E). Although the baseline levels of H2B_ub_ were significantly suppressed after actinomycin D, increasing signal intensity revealed that there was still a significant increase in H2B_ub_ above the unirradiated control at 3 to 4 hours after treatment with high-LET protons. H2B_ub_ induction by high-LET protons was also shown quantitatively in two head and neck squamous cell carcinoma cells (UMSCC74A and UMSCC6), suggesting that H2B_ub_ is an epigenetic marker of CDD sites (Fig. E2A-D, available online at www.redjournal.org).

### High-LET protons directly cause persistent CDD

**Fig. 4:**
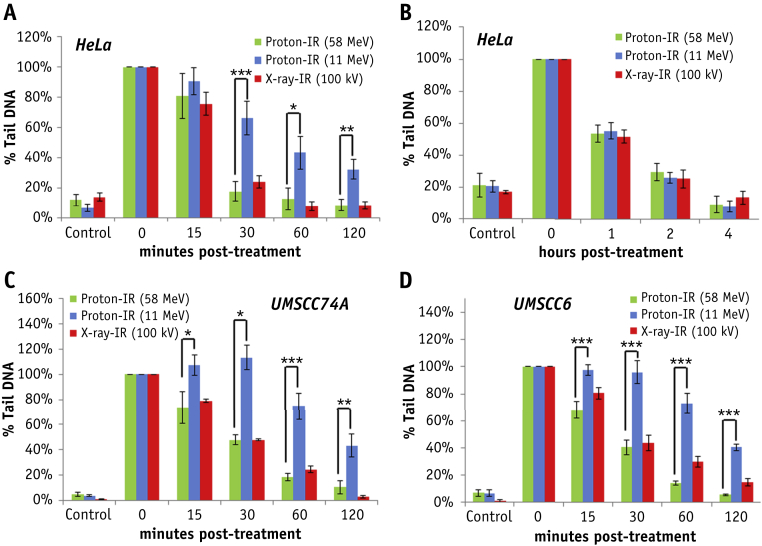
High linear energy transfer (LET) protons induce DNA single-strand breaks (SSBs) and alkali-labile site-associated DNA damage, which displays slow repair kinetics. (A) HeLa, (C) UMSCC74A, or (D) UMSCC6 cells were irradiated with 1.5 Gy X rays or protons at low or high LET and SSBs/alkali labile sites measured at various time points after ionizing radiation (IR) by the alkaline comet assay. (B) HeLa cells were irradiated with 4 Gy IR and DNA double-strand breaks measured at various time points after IR by the neutral comet assay. Shown is the percent tail DNA ± standard deviation normalized to the levels seen immediately after IR, which was set to 100%. *P < .005, **P < .002, ***P < .001, as analyzed by a one-sample *t* test.

**Fig. 5:**
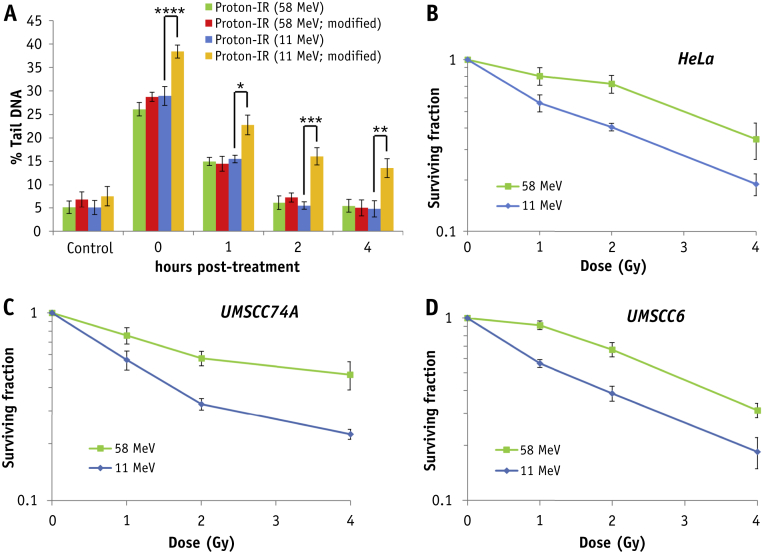
High linear energy transfer (LET) protons directly induce persistent complex DNA damage (CDD) formation, which contributes to increased radiosensitivity. (A) HeLa cells were irradiated with 4 Gy protons at low or high LET and CDD measured at various time points after ionizing radiation (IR) by the enzyme-modified neutral comet assay in the absence or presence (indicated as modified) of APE1, NTH1, and OGG1. Shown is the percent tail DNA ± standard deviation. *P < .02, **P < .01, ***P < .005, ****P < .001, as analyzed by a one-sample *t* test. Clonogenic survival of (B) HeLa, (C) UMSCC74A, and (D) UMSCC6 cells after protons at low or high LET. Shown is the surviving fraction ± standard error. A comparison of the surviving fraction at 2 Gy (SF2) is statistically significantly different by one-way analysis of variance (*P* < .02 for HeLa and UMSCC74A; *P* < .01 for UMSCC6).

Using the alkaline comet assay, detecting SSBs and alkali-labile sites, HeLa cells repair the DNA damage induced by X rays and low-LET protons within approximately 30-60 minutes after treatment. In contrast, high-LET protons generate DNA damage that persists up to 2 hours after irradiation, suggesting CDD formation (Fig. 4A). Interestingly the repair of DSBs detected by the neutral comet assay was the same under all IR conditions (Fig. 4B). These data were also reproduced in UMSCC74A and UMSCC6 (Fig. 4C, D and Fig. E3A, B [available online at www.redjournal.org]). The existence of CDD induced by high-LET protons was proven using the enzyme-modified neutral comet assay, using recombinant DNA repair enzymes (APE1, OGG1, and NTH1) to incise residual DNA base damage and abasic sites. Therefore, whereas there was no difference in the kinetics of repair of DSBs in HeLa cells induced by low-LET or high-LET protons after mock treatment (Fig. 5A, green and blue bars), only cells irradiated with high-LET protons caused a significant elevation in strand breaks immediately after irradiation following enzyme addition, which persisted 2 to 4 hours after irradiation (Fig. 5A, yellow bars). As a consequence, using clonogenic survival assays we demonstrate that HeLa cells show significantly decreased cell survival after high-LET protons versus low-LET protons (Fig. 5B; RBE = 1.67 ± 0.14). Similarly we observed the same significant LET-dependent difference in survival of UMSCC74A and UMSCC6 cells after proton irradiation (Fig. 5C, D; RBE = 1.85 ± 0.15 and 1.68 ± 0.10, respectively). The α values are shown in Table E1 and fitted survival curves in Fig. E4A-C (both available online at www.redjournal.org).

### MSL2 and RNF20/40 catalyze H2B_ub_ that signals CDD and its repair

**Fig. 6:**
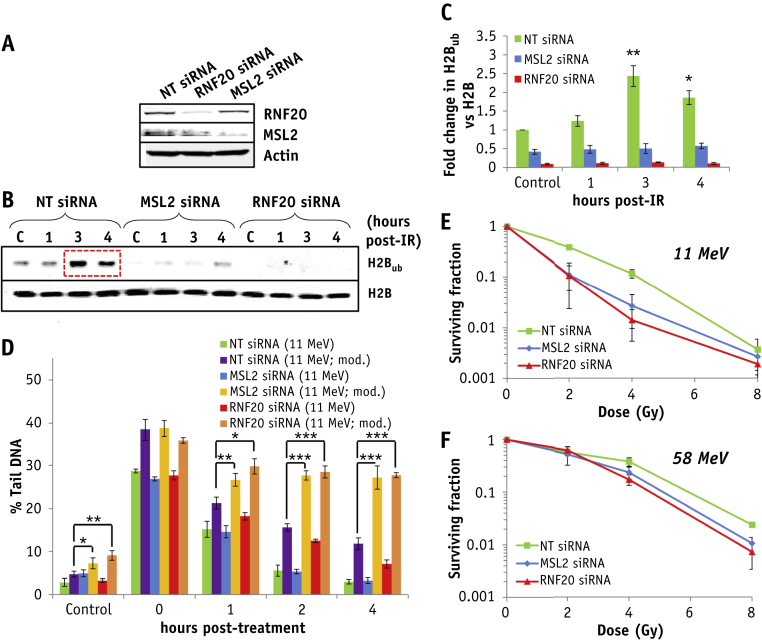
RNF20 and MSL2 catalyze formation of H2B_ub_ in response to complex DNA damage (CDD) induced by high linear energy transfer (LET) protons. (A-F) HeLa cells were treated with non-targeting control siRNA (NT), MSL2, or RNF20 small interfering RNA (siRNA). (A) Whole cell extracts were analyzed by immunoblotting. (B, C) Cells were untreated (designated C) or irradiated with 10 Gy high-LET protons and harvested at the time points indicated after treatment and purified histones analyzed by immunoblotting. (C) Shown is the mean fold change ± standard deviation in H2B_ub_ normalized against H2B. *P < .02, **P < .005, as analyzed by a one-sample *t* test of fold change in H2B_ub_ versus the unirradiated control. Red box indicates increased levels of H2B_ub_. (D) HeLa cells were irradiated with 4 Gy protons at high LET and CDD measured at various time points after ionizing radiation by the enzyme-modified neutral comet assay after incubation in the absence or presence (as indicated by “mod”) of APE1, NTH1, and OGG1. Shown is the percent tail DNA values ± standard deviation. *P < .02, **P < .005, ***P < .001, as analyzed by a one-sample t-test. (E, F) Clonogenic survival of HeLa cells was analyzed after protons at (E) high LET and (F) low LET. Shown is the surviving fraction ± standard error. A comparison of the surviving fraction at 2 Gy (SF2) after NT siRNA versus MSL2 (*P* < .01) or RNF20 (*P* < .05) siRNA only after high-LET protons is statistically significantly different by one-way analysis of variance.

RNF20/40 is well known to catalyze H2B_ub_, additionally MSL2 has been shown to catalyze histone H2B ubiquitylation, albeit on lysine 34 (25). We therefore assessed the roles of these enzymes in CDD signaling and repair. An siRNA knockdown of RNF20 (Fig. 6A) caused a significant reduction (approximately 90%) of H2B_ub_ in unirradiated HeLa cells, but also suppressed induction of H2B_ub_ 3 to 4 hours after irradiation with high-LET protons, versus a non-targeting control siRNA (Fig. 6B, C). Surprisingly, depletion of MSL2 similarly reduced (approximately 60%) unirradiated levels of H2B_ub_ and those in response to high-LET protons, suggesting that both RNF20/40 and MSL2 are involved in stimulating H2B_ub_ in response to CDD. An siRNA knockdown of RNF20, but not MSL2, caused a delay in the repair of DSBs induced by both high-LET and low-LET protons (Fig. E5A and E5B, available online at www.redjournal.org), consistent with its proposed role in modulating DSB repair 16, 17. These data using high-LET protons were also replicated using the enzyme-modified neutral comet assay following mock treatment of cells (Fig. 6D, green, blue, and red green bars). However, addition of recombinant repair enzymes (APE1, OGG1 and NTH1) to measure CDD caused an increase in strand breaks in unirradiated cells after RNF20 or MSL2 siRNA compared with non-targeting control siRNA-treated cells but also caused a significant persistence of CDD up to 4 hours after high-LET protons (Fig. 6D, purple, yellow, and orange bars). Furthermore, depletion of RNF20 and MSL2 significantly increased sensitivity of cells to high-LET protons versus the non-targeting control siRNA (Fig. 6E). In contrast, depletion of these enzymes did not alter radiosensitivity with low-LET protons where significantly less CDD is induced (Fig. 6F).

## Discussion

CDD is a signature of IR thought to contribute significantly to cell killing, owing to the difficult nature of its repair, and is an important factor contributing to effective cancer treatment by radiotherapy. High-LET IR, such as α-IR, is more efficient at producing CDD than low-LET X rays or γ-IR, and there is increasing evidence that persistent DNA damage, predictably CDD, can be generated after low-energy (high-LET) protons 10, 11, 12. CDD is less efficiently repaired than isolated DNA damage, as revealed by persistence of the damage but also of DNA repair protein foci (26). Despite this, the radiobiology and the cellular response to CDD in chromatin has not previously been identified.

On screening of site-specific histone PTMs after high- and low-LET IR, therefore varying the frequency of CDD produced, we discovered surprisingly that the majority of these PTMs do not change dramatically over a time course after IR, similar to a previous study examining phleomycin to generate DSBs (18). Only a decrease in the mitotic markers H3 phosphorylation on serine 10 and 28 at later time points after irradiation, irrespective of the treatment, was observed. Furthermore, no significant changes in cell cycle position were observed after irradiation with high- or low-LET protons. Focusing on CDD-specific PTMs, only H2B ubiquitylation at lysine 120 (H2B_ub_) was consistently induced after both high-LET α-IR and protons at >2 hours after irradiation when CDD persists. Interestingly, high-LET protons also appeared to promote a decrease in acetylation of H3K14 and increases in trimethylation of H3K9, H3K36, and H3K79 at later time points after IR, which require further investigation.

H2B_ub_ has previously been suggested to be induced >1 hour after treatment in response to DSBs by the radiomimetic neocarzinostatin and X rays irradiation 16, 17, although another study showed H2B_ub_ levels do not alter after neocarzinostatin (27). In agreement with the latter, we found no evidence for an elevation of H2B_ub_ in response to DSBs induced by low-LET radiation, including γ-IR, X rays, and low-LET protons, particularly 2 hours after IR when the majority of DSBs have been repaired. Instead we demonstrate that H2B_ub_ is specifically induced by high-LET α-IR and protons, at approximately 2 to 6 hours after irradiation, which correlates with the slow kinetics of CDD repair, as revealed by enzyme-modified neutral comet assays. We were unable to demonstrate any difference in the repair of DSBs using neutral comet assays after low- versus high-LET protons, whereas there was a persistence of SSBs/alkali-labile sites using alkaline comet assays after high-LET protons, suggesting that CDD sites are non-DSB in nature. In support of this, levels of non-DSB-associated CDD were shown to be 4 times higher than the levels of DSBs after low-LET IR (28) but predicted to be 8 times higher after high LET (5).

Intriguingly, we discovered that H2B_ub_ induced by CDD is catalyzed by 2 E3 ubiquitin ligases, RNF20/40 and MSL2. A role for RNF20/40 in response to DSBs has previously been reported 16, 17, 27, and indeed we observed a deficiency in the repair of DSB induced by both low- and high-LET protons after RNF20 depletion, but arguably that induction of H2B_ub_ is not essential for this process. We also demonstrate that deletion of RNF20 leads to almost a complete reduction in H2B_ub_, as observed in the previous studies highlighted above, largely because this histone mark is involved in co-ordination of DNA transcription. Additionally, however, we now propose a role for RNF20/40 in the signaling and repair of CDD induced by high-LET radiation. Interestingly we discovered that CDD repair is also dependent on MSL2. It has been proposed that MSL2 targets ubiquitylation of H2B on lysine 34 for transcriptional activation (25) but that a knockdown of MSL2 causes a significant reduction in H2B_ub_ by decreasing the association of RNF20/40 with chromatin. A follow-up study demonstrated that MSL1/2 and RNF20/40 directly interact and are dependent on each other for stabilization on chromatin in association with transcription elongation factors (29). Similarly, we observed a substantial reduction in H2B_ub_ in the absence of MSL2 and are in support of the evidence that MSL2 and RNF20/40 are mutually dependent in co-ordinating H2B_ub_. However, we now advance these findings further to describe that these enzymes are crucial for the co-ordination and efficiency of IR-induced CDD repair and are important factors responsive to high-LET radiation.
