# Supplementary material for: Complex DNA Damage Induced by High Linear Energy Transfer Alpha-Particles and Protons Triggers a Specific Cellular DNA Damage Response
Source: Int J Radiat Oncol Biol Phys. 2018 Mar 1;100(3):776–84. doi: 10.1016/j.ijrobp.2017.11.012 (PMC5796827; doi:10.1016/j.ijrobp.2017.11.012)
Supplement: Supplementary Information [file mmc1.docx]

**Supplementary methods**

**Antibodies and proteins**

Details of all antibodies are included in the table below. Recombinant histagged NTH1, OGG1 and APE1 were overexpressed in Rosetta2(DE3)pLysS bacterial cells, and purified by HisTrap chromatography (GE Healthcare, Little Chalfont, UK) using a gradient elution of imidazole and an AKTA purifier FPLC system. siRNA sequences targeting RNF20 and MSL2 (both SMARTpool siGENOME; GE Healthcare, Little Chalfont, UK) are as follows: RNF20/40 (Cat No. M-007027-00) : 5'-CCAAUGAAAUCAAGUCUAA-3', 5'-UAAGGAAACUCCAGAAUAU-3', 5'-GCAAAUGUCCCAAGUGUAA-3', 5'-AGAAGAAGCUACAUGAUUU-3'; MSL2 (Cat No. M-020828-01): 5'-GCAGUUCUGUUAUCAAUGG-3', 5'-UCUCUUAGCCAUAAUGUUU-3', 5'-CCAGUACACAUGAUGAUAA-3', 5'-GAGUAUAUAACACAGACUA-3'. The non-targeting control siRNA (AllStars Negative Control siRNA, Cat No. SI02655450; Qiagen, Manchester, UK) was used although the sequence is proprietary information.

| **ANTIBODY** | **COMPANY** | **CATALOGUE NUMBER** |
| --- | --- | --- |
| Anti-ubiquityl-Histone H2A (K119) | Merck-Millipore | 05-678 |
| Anti-ubiquityl-Histone H2AX (K119) | Merck-Millipore | AB10029 |
| Anti-ubiquityl-Histone H2B (K120) | MediMabs | AB10029 |
| Anti-phospho-Histone H2AX (S139) | Merck-Millipore | 05-636 |
| Anti-acetyl-Histone H2B (K5) | Cell Signaling Technology | 2574 |
| Anti-acetyl-Histone H2B (K12) | Cell Signaling Technology | 9861 |
| Anti-acetyl-Histone H2B (K15) | Cell Signaling Technology | 9083 |
| Anti-acetyl-Histone H2B (K20) | Cell Signaling Technology | 2571 |
| Anti-acetyl-Histone H4 (K5) | Cell Signaling Technology | 8647 |
| Anti-acetyl-Histone H4 (K8) | Cell Signaling Technology | 2594 |
| Anti-acetyl-Histone H4 (K12) | Cell Signaling Technology | 13944 |
| Anti-phospho-Histone H3 (S10) | Cell Signaling Technology | 3377 |
| Anti-phospho-Histone H3 (S28) | Cell Signaling Technology | 9713 |
| Anti-acetyl-Histone H3 (K9) | Cell Signaling Technology | 9649 |
| Anti-acetyl-Histone H3 (K14) | Cell Signaling Technology | 4318 |
| Anti-acetyl-Histone H3 (K18) | Cell Signaling Technology | 9675 |
| Anti-acetyl-Histone H3 (K27) | Cell Signaling Technology | 4353 |
| Anti-acetyl-Histone H3 (K56) | Cell Signaling Technology | 4243 |
| Anti-trimethyl-Histone H3 (K9) | Cell Signaling Technology | 13969 |
| Anti-trimethyl-Histone H3 (K27) | Cell Signaling Technology | 9733 |
| Anti-trimethyl-Histone H3 (K36) | Cell Signaling Technology | 4909 |
| Anti-trimethyl-Histone H3 (K79) | Cell Signaling Technology | 4260 |
| Anti-dimethyl-Histone H3 (K4) | Cell Signaling Technology | 9725 |
| Anti-dimethyl-Histone H3 (K9) | Cell Signaling Technology | 4658 |
| Anti-dimethyl-Histone H3 (K27) | Cell Signaling Technology | 9728 |
| Anti-dimethyl-Histone H3 (K36) | Cell Signaling Technology | 2901 |
| Anti-Histone H3 | Cell Signaling Technology | 2935 |
| Anti-Histone H4 | Cell Signaling Technology | 3638 |
| Anti-Histone H2B | Santa Cruz | sc-10808 |
| Anti-RNF20 | Bethyl Labs | A300-714 |
| Anti-MSL2 | Aviva Systems Biology | ARP43246 |
| IRDye 800CW goat anti-mouse IgG | Li-Cor Biosciences | 926-32210 |
| IRDye 800CW goat anti-rabbit IgG | Li-Cor Biosciences | 926-32211 |
| Alexa Fluor 680 goat anti-mouse IgG | Life Technologies | A-21057 |
| Alexa Fluor 680 goat anti-rabbit IgG | Life Technologies | A-21076 |

**Histone extractions**

Cell pellets were resuspended in hypotonic buffer (10 mM Tris-HCl, pH 8.0, 1 mM KCl, 1.5 mM MgCl_2_) containing 1 µg/ml pepstatin, leupeptin, chemostatin and aprotinin, 1 mM N-ethylmaleimide and 0.1 mM PMSF, and incubated for 30 min at 4°C with shaking. Nuclei were pelleted by centrifugation (), resuspended in 0.4 M sulfuric acid and incubated overnight at 4°C with shaking. Samples were centrifuged at 16,000 x g for 10 min at 4°C, the supernatant containing histones removed and precipitated with trichloroacetic acid (33 %) on ice for 30 min. Histones were pelleted (16,000 x g for 10 min at 4°C), washed twice with ice-cold acetone and air dried at room temperature. Histone pellets were redissolved in water, concentrations measured using a Nanodrop (Thermo Scientific, Warrington, UK) before adding 3x SDS-PAGE loading buffer (25 mM Tris-HCl, pH 6.8, 2.5 % β-mercaptoethanol, 1 % SDS, 10 % glycerol, 1 mM EDTA, 0.05 mg/ml bromophenol blue). Histones (~1 µg) were separated by 16 % Tris-glycine SDS-PAGE and transferred onto an Immobilon FL PVDF membrane (Millipore, Watford, UK). Membranes were blocked using Odyssey blocking buffer (Li-cor Biosciences, Cambridge, UK) and incubated with the primary antibody overnight at 4°C. Membranes were washed with PBS containing 0.1 % Tween 20, incubated with either Alexa Fluor 680 or IR Dye 800 secondary antibodies for 1 h at room temperature and further washed with PBS containing 0.1 % Tween 20. Histones were visualized and quantified using the Odyssey image analysis system (Li-cor Biosciences, Cambridge, UK).

**Cell cycle analysis**

Cells were trypsinised, washed twice with ice cold PBS (100 x g for 5 min at 4°C), fixed with ice cold 70 % ethanol and kept at 4°C until analysis. Fixed cells were centrifuged (200 x g for 5 min at 4°C), washed with PBS containing 0.05 % Tween-20, and then resuspended in PBS containing 0.05 % Tween-20, 10 µg/ml propidium iodide and 0.1 mg/ml RNase A for 1 h at room temperature. Cell cycle analysis was performed by fluorescence-activated cell sorting (FACS) using the Attune NxT Flow Cytometer (Life Technologies, Paisley, UK).

**Single cell gel electrophoresis (Comet) assays.**

The alkaline comet assay for measurement of DNA single strand breaks and alkali labile sites was performed as follows. Briefly cells were trypsinised, diluted to 1 × 10^5^ cells/ml and 250 µl aliquots of the cell suspension placed into the wells of a 24 well plate which was placed on ice. Cells were irradiated (1.5 Gy) and embedded on a microscope slide in low melting agarose (Bio-Rad, Hemel Hempstead, UK). The slides were incubated for up to 2 h at 37°C in a humidified chamber to allow for DNA repair, prior to lysis in buffer containing 2.5 M NaCl, 100 mM EDTA, 10 mM Tris-HCl pH 10.5, 1 % (v/v) DMSO and 1 % (v/v) Triton X-100 for 1 h at 4°C. The slides were then incubated in the dark for 30 min in cold electrophoresis buffer (300 mM NaOH, 1 mM EDTA, 1 % (v/v) DMSO, pH 13) to allow the DNA to unwind, prior to electrophoresis at 25 V, 300 mA for 25 min. Slides were neutralised with three 5 min washes of 0.5 M Tris-HCl (pH 8.0) and allowed to dry overnight. The neutral comet assay for measurement of DNA double strand breaks was similar to that described above, but with the following modifications. Cells were irradiated (4 Gy) and slides were incubated for up to 4 h to allow for DNA repair. Cell lysis was performed in buffer containing 2.5 M NaCl, 100 mM EDTA, 10 mM Tris-HCl pH 10.5, 1 % N-lauroylsarcosine, 1 % DMSO and 1 % (v/v) Triton X-100. Electrophoresis was performed in cold 1 × TBE buffer (pH 8.3) at 25 V, ~20 mA for 25 min. Finally slides were washed three times with 1 × PBS before allowing to dry overnight. For detection of complex DNA damage, an enzyme modified neutral comet assay was used as follows. In brief, the neutral comet assay was followed as described above but following cell lysis, slides were washed three times with enzyme reaction buffer (40 mM HEPES-KOH, 100 mM KCl, 0.5 mM EDTA and 0.2 mg/ml BSA, pH 8.0). Slides were then incubated with either buffer alone (mock treated) or with buffer containing 5 pmol OGG1, 6 pmol NTH1 and 0.6 pmol APE1 for 1 h at 37°C in a humidified chamber. These enzymes were prepared by overexpression in Rosetta2(DE3)pLysS bacterial cells using the pET28a plasmids (kindly provided by G. Dianov) and purified by HisTrap chromatography (GE Healthcare, Little Chalfont, UK) using a gradient elution of imidazole and an AKTA purifier FPLC system. Slides were washed three times with cold 1 × TBE buffer and electrophoresed in the same buffer prior to washing with 1 × PBS, as described above. Once dried, all slides from both alkaline, neutral and enzyme modified comets were subsequently rehydrated for 30 min in water (pH 8.0), stained for 30 min with SYBR Gold (Life Technologies, Paisley, UK) diluted 1:10,000 in water (pH 8.0) and again dried overnight. Cells (50 per slide, in duplicate) were analysed from the dried slides using the Komet 6.0 image analysis software (Andor Technology, Belfast, Northern Ireland) and % tail DNA values averaged from at least three independent experiments.

**Supplementary Table**

**Table E1.** Survival curve characteristics for cells following proton irradiation. Data from survival curves were fitted to the equation ln(SF)=-αD, where D equals dose and SF is surviving fraction, and α values are shown below.

| **Cell Line** | **α (Gy^-1^) – 58 MeV** | **α (Gy^-1^) – 11 MeV** |
| --- | --- | --- |
| HeLa | 0.26±0.03^a^ | 0.44±0.02 |
| UMSCC74A | 0.22±0.03 | 0.41±0.03 |
| UMSCC6 | 0.27±0.02 | 0.45±0.03 |

^a^Mean±SE

**Supplementary figures**

**
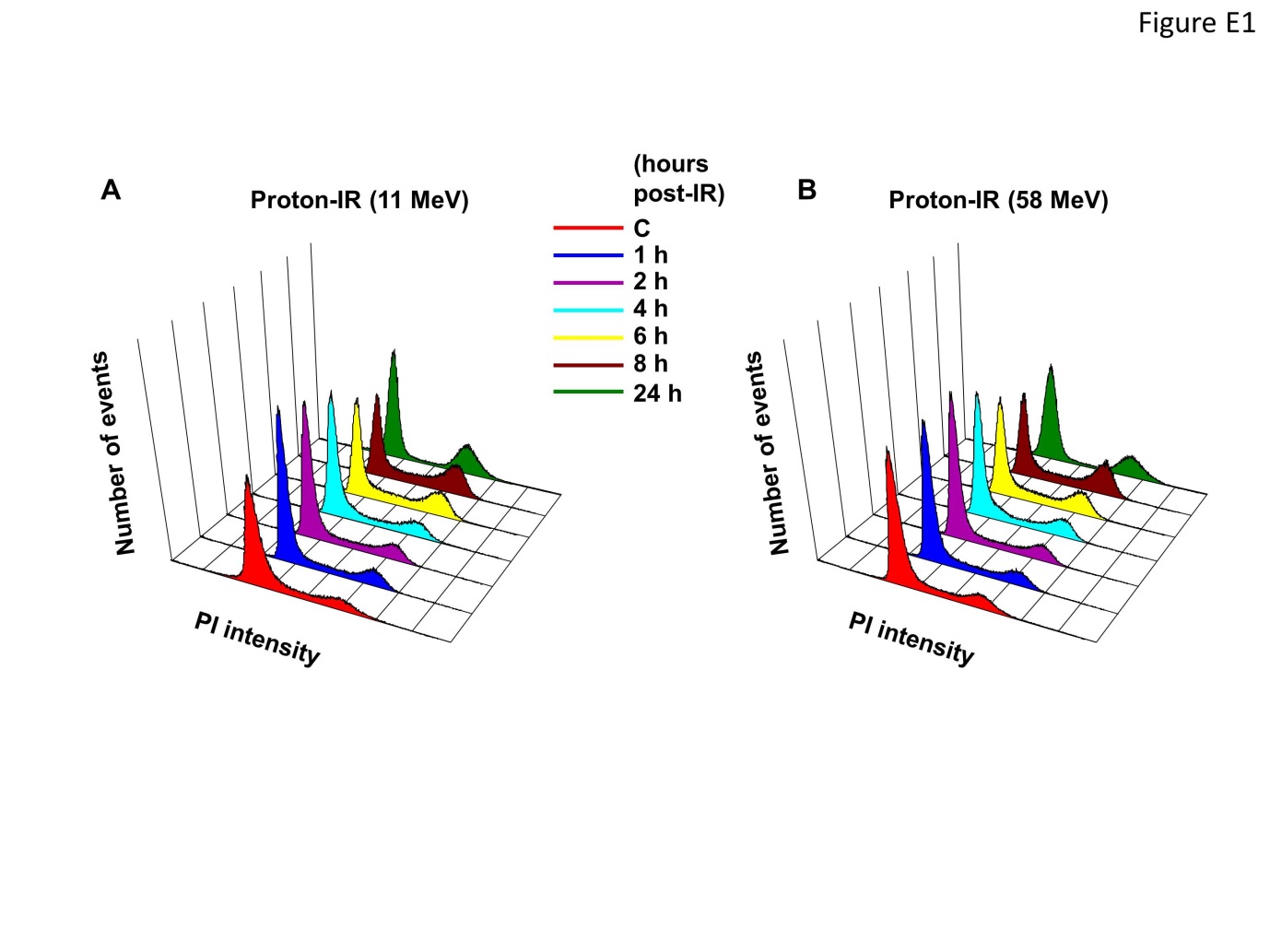
**

**Figure E1.** Cells display similar cell cycle profiles post-irradiation with either low- and high-LET protons. HeLa cells were unirradiated (designated C) or irradiated with 4 Gy protons at (A) high-LET (11 MeV) or (B) low-LET (58 MeV) and harvested at the time points indicated (1-24 h) post-treatment. Cell cycle profiles were determined by FACS analysis.

**
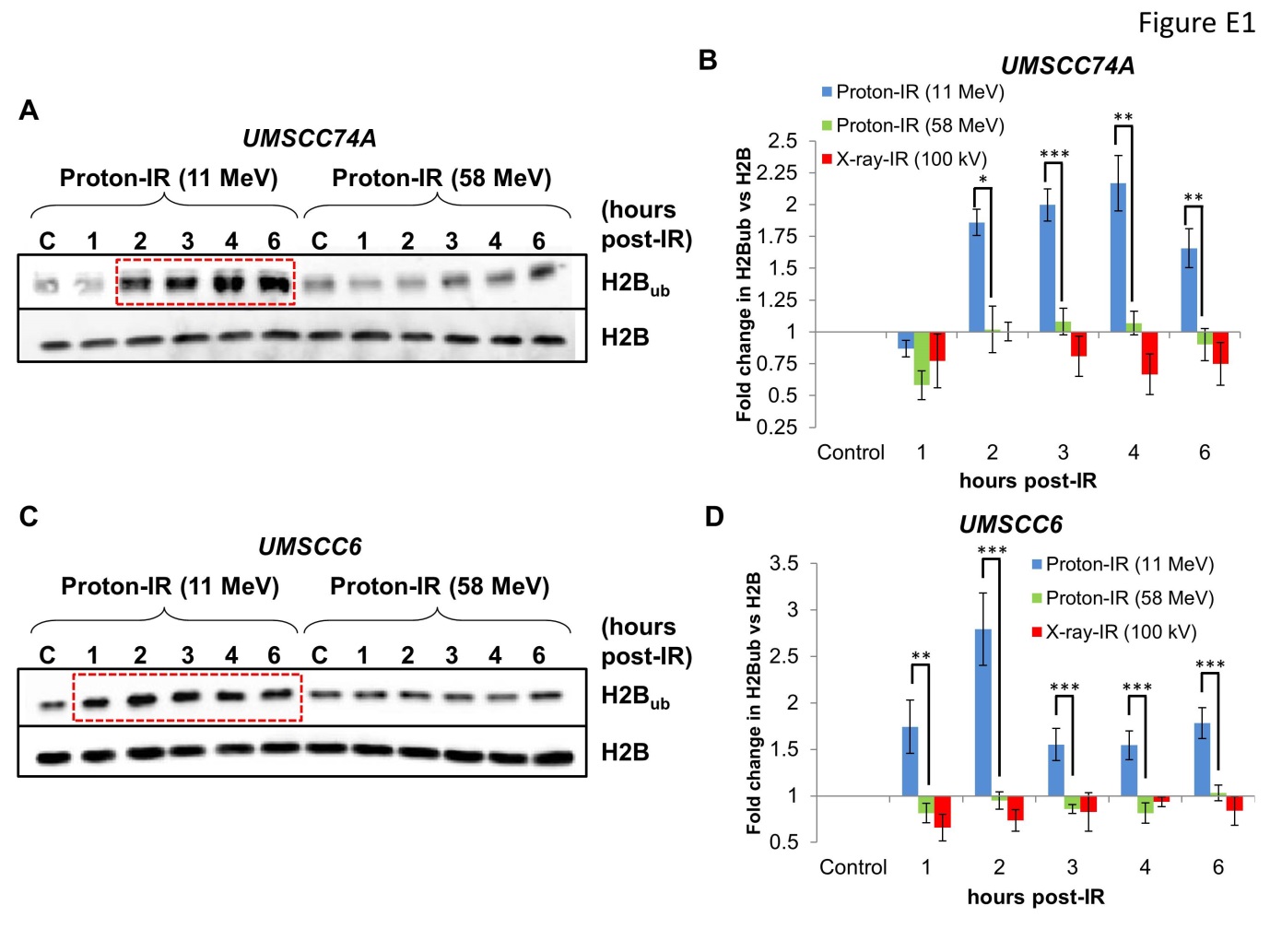
**

**Figure E2.** H2B_ub_ is induced in head and neck squamous cell carcinoma cells in response to CDD-induced by high-LET proton irradiation. (A-D) UMSCC74A and UMSCC6 cells were untreated (designated C) or irradiated with 10 Gy protons at low-LET (58 MeV) or high-LET (11 MeV) or 10 Gy x-rays (100 kV), and harvested at the time points indicated (1-6 h) post-treatment. Histones were purified by acid extraction, separated by 16 % SDS-PAGE and analysed by immunoblotting using the indicated antibodies. Red boxes indicate increased levels of H2B_ub_. (B and D) Shown is the mean fold change in H2B_ub_ normalised against H2B, with standard deviations from at least three independent experiments. *p<0.05, **p<0.02, ***p<0.005 as analysed by a two sample *t*-test of fold increase in H2B_ub­_ values following low-LET versus high-LET protons at each particular time point.

**
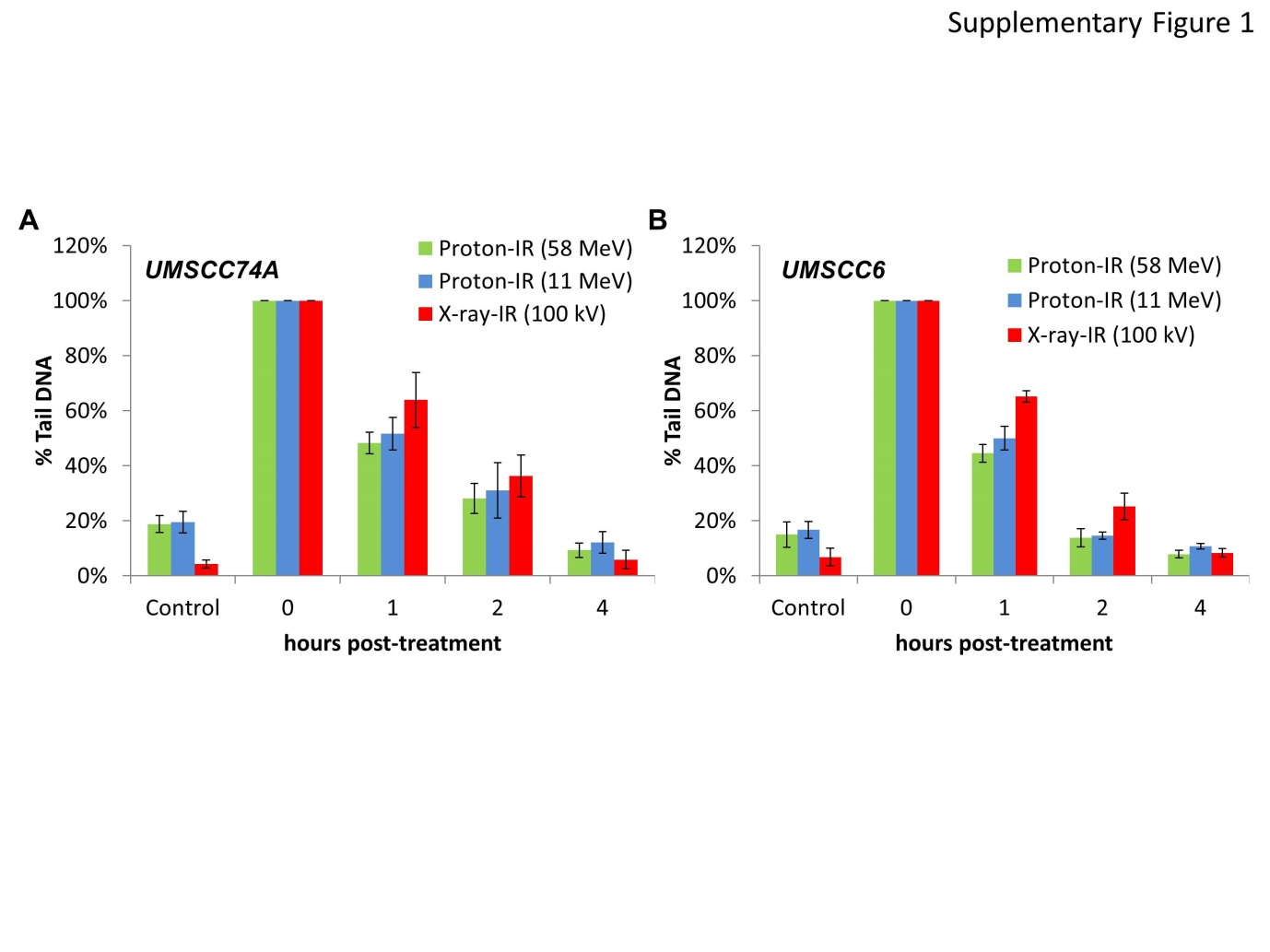
**

**Figure E3.** Low- and high-LET protons induce DSB which display similar DNA repair kinetics. (A) UMSCC74A or (B) UMSCC6 cells were irradiated with 4 Gy protons at low-LET (58 MeV) or high-LET (11 MeV) and DNA DSBs measured at various time points post-IR by the neutral comet assay. Shown is the % tail DNA with standard deviations from at least three independent experiments normalised to the levels seen immediately post-IR (0 min) which was set to 100 %.

**
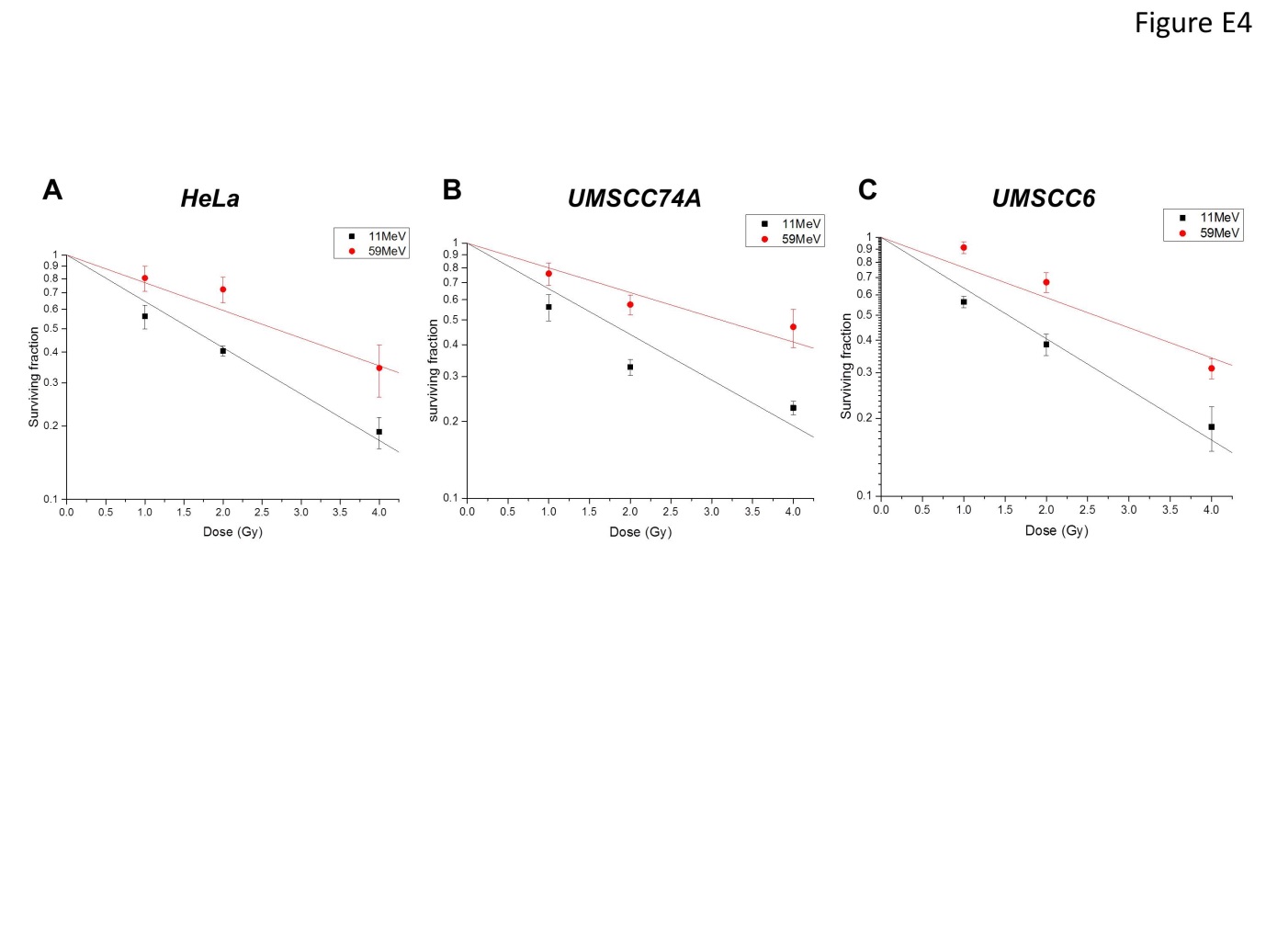
**

**Figure E4.** High-LET protons display increased radiosensitivity compared to low-LET protons. Clonogenic survival data of (A) HeLa, (B) UMSCC74A and (C) UMSCC6 cells following protons at low-LET (58 MeV) or high-LET (11 MeV) are shown. Data were fitted to the equation ln(SF)=-αD, where D equals dose and SF is surviving fraction using OriginPro 9.1.

**
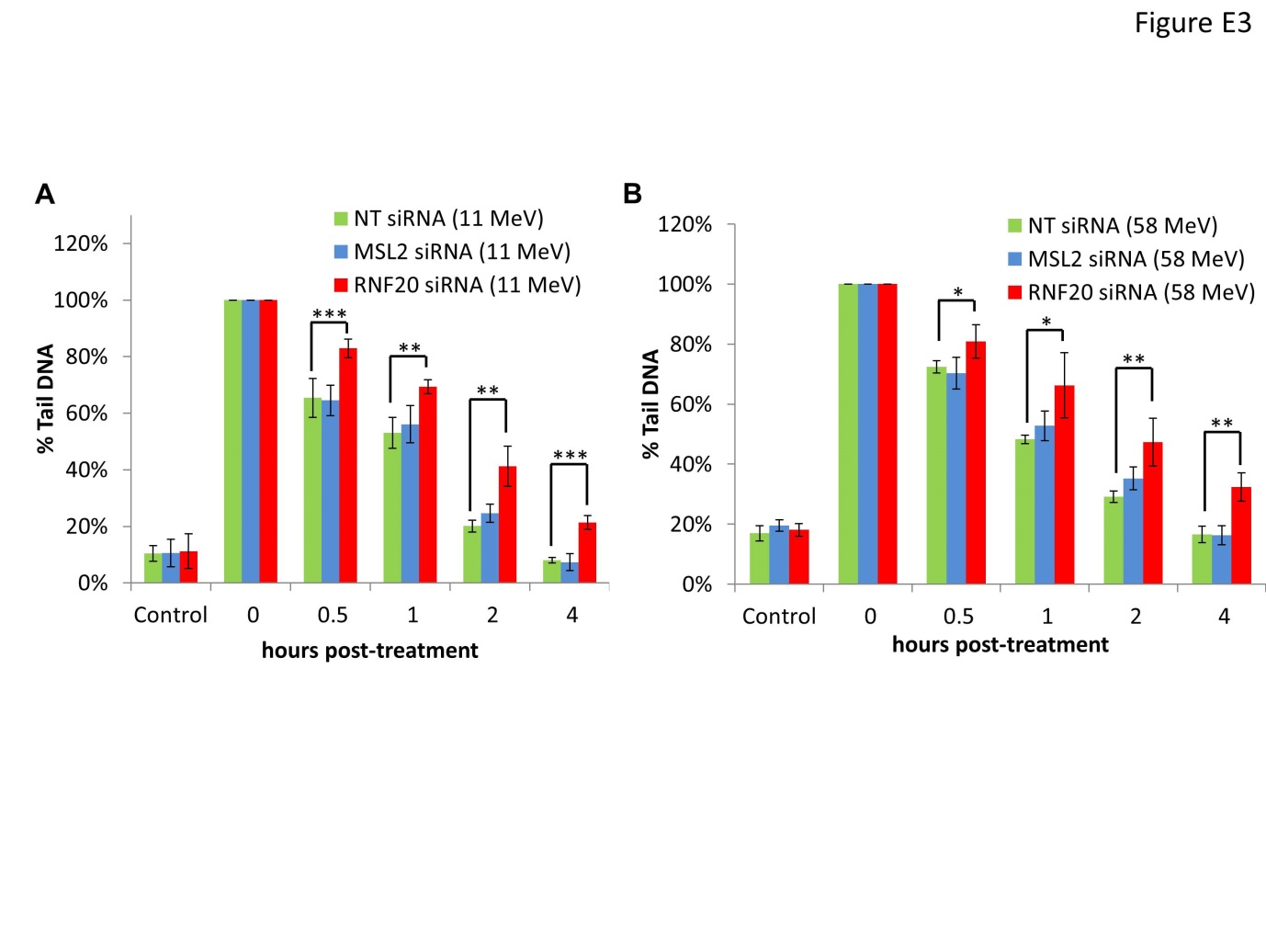
**

**Figure E5.** Depletion of RNF20/40 causes a delay in the repair of DSBs generated by either low-LET or high-LET protons. (A-B) HeLa cells were treated with non-targeting control siRNA, MSL2 or RNF20 siRNA for 48 h. Cells were irradiated with 4 Gy protons at (A) high-LET (11 MeV) or (B) low-LET (58 MeV) and DNA DSBs measured at various time points post-IR by the neutral comet assay. Shown is the % tail DNA with standard deviations from at least three independent experiments normalised to the levels seen immediately post-IR (0 min) which was set to 100 %. *p<0.05, **p<0.02, ***p<0.01 as analysed by a one sample *t*-test of % tail DNA following RNF20 siRNA treatment in comparison to the non-targeting control siRNA at each particular time point.
